# A Hitchhiker’s Ride: The Honey Bee Louse *Braula Coeca* (Diptera: Braulidae) Selects its Host by Eavesdropping

**DOI:** 10.1007/s10886-024-01481-2

**Published:** 2024-02-23

**Authors:** Abdullahi Yusuf, Christian Pirk, Robin Crewe

**Affiliations:** Social Insects Research Group, Department of Zoology and Entomology, University of Pretoria, Private Bag X20, Hatfield, 0028 Republic of South Africa

**Keywords:** Honey bee parasite, Kairomones, Mandibular gland secretions, Cuticular hydrocarbons, Trophallaxis

## Abstract

The bee louse *Braula spp.* had until recently a distribution coincident with its host the honey bee. The adult fly usually attaches to a worker honey bee and steals food from its mouth. However, not all worker bees carry *Braula spp.* and the mechanism used by *Braula spp.* to select hosts is not well understood. Using choice remounting bioassays and chemical analyses, we determined host selection and the cues used by *B. coeca*, a species associated with the African honey bee *Apis mellifera scutellata*. *Braula coeca* successfully remounted bees from which they were initially removed and preferred their mandibular gland pheromones (MDG) over those of bees not carrying them. The bee lice did not show any preference for the cuticular hydrocarbons of both types of workers. Chemical analyses of the MDG extracts, revealed quantitative differences between the two categories of workers, with workers carrying *B. coeca* having more of the queen substance (9-oxo-2(*E*)-decenoic acid) and worker substance (10-hydroxy-2(*E*)-decenoic). *Braula coeca* showed a dose response to the queen substance, indicating its ability to use host derived kairomones as cues that allowed it to benefit from trophallactic dominance by individuals that have a higher probability of being fed by other workers.

**Supplementary Information:**

The online version contains supplementary material available at 10.1007/s10886-024-01481-2.

## Introduction

*Braula spp.* known as the bee lice, are small (~ 1.6 mm) wingless flies that have a close association with honey bees (Hepburn 1978). The genus *Braula* contains five species (namely *B. coeca, B. schmitzi, B. orientalis, B. pretoriensis, B. kohli*), and had a global distribution (Smith and Caron 1984). But it is now restricted (Kulincevic et al. 1991) to regions where they are considered as having negligible threat to honey bees, or places where miticides are not used to treat *Varroa* infestations (Zaitoun and Al-Ghzawi 2008; Gidey et al. 2012; Strauss et al. 2014; Rodrigues and Serrano 2019; Esnault et al. 2019; Martin and Bayfield 2014; Zapata-Carvajal et al. 2017). The adult fly has a long-term commensal association with honey bees where it usually attaches itself to a honey bee (Büscher et al. 2022) and steals food (reviewed in Weems and Sanford 2006) by inducing regurgitation through striking the upper end of the bee’s labium until it extends its tongue (Esnault et al. 2019). Opinions differ regarding the status of the adult *Braula spp.* as a parasite, with some maintaining that, it causes little or no harm to bee colonies apart from consuming honey and pollen stores (Hepburn 1978). While others see *Braula spp.* as harmful parasites of great concern for local beekeepers (Crane 1990; Zaitoun and Al-Ghzawi 2008). This is because, tunnels built within honey cells by developing fly maggots disfigure honey combs, thus affecting the quality of comb honey (Hepburn 1978; Shimanuki et al. 1999). Heavy maggot infestations especially in weak colonies causes paralysis of larvae and decreases the queen’s egg laying efficiency (Kessler 1987); death of developing bees could also occur (Marcangeli et al. 1993). However, not all bees in a hive attract or carry the bee lice, because it prefers the queen, nurse bees and rarely attaches to drones (Smith and Caron 1984). The underlying mechanism that influences the attraction of *Braula spp.* to queens and nurse worker bees is not well known except for the fact that, they respond to honey bee pheromones (Kaschef 1959) and*,* uses chemical camouflage to survive in the hive (Martin and Bayfield 2014).

The honey bee colony provides an ample variety of cues from temperature caused by heating workers in the brood nest (Basile et al. 2008) to brood pheromones emitted by the open brood (Le Conte et al. 1990, 2006) and to mandibular gland pheromones relating to the reproductive activity level of the emitter (Crewe and Velthuis 1980). Since adult *Braula spp.* feed on the food exchanged during trophallactic interactions and reproductively dominant individuals receive more food from other workers (Korst and Velthuis 1982), thus *Braula spp.* should prefer reproductively dominant honey bee hosts, such as the queen or workers that smell like or act like a queen, since these individuals receive more food than their non-reproductive counterparts. Reproductive dominance is normally associated with pheromonal dominance (Crewe and Velthuis 1980; Zheng et al. 2010), the latter allowing the dominant worker to be fed by others so as to activate its ovaries following the social pathway (Schäfer et al. 2006). Therefore, it would be beneficial for the fly to detect differences between individuals within a honey bee colony in order to identify a host worker that has a higher likelihood of receiving food through trophallaxis. Therefore, we hypothesized that *Braula spp.* would be attached to those worker bees that are pheromonally distinct and produce pheromone profiles similar to those of a queen, increasing the chance of attaching to individuals that will receive food. To test this, we conducted re-mounting bioassays offering the choice between bees carrying and those not carrying *B. coeca* to the lice, analyzed the cuticular hydrocarbon profiles of bees and those of *B. coeca* as well as, pheromones from the mandibular glands of worker bees to determine the possible sources of cues used by *B. coeca.* Finally, we conducted bioassays using the chemical cues to determine which ones are used by *Braula spp.* for host selection within the colony.

## Materials and Methods

### Honey Bees and Bee Lice

**Fig. 1:**
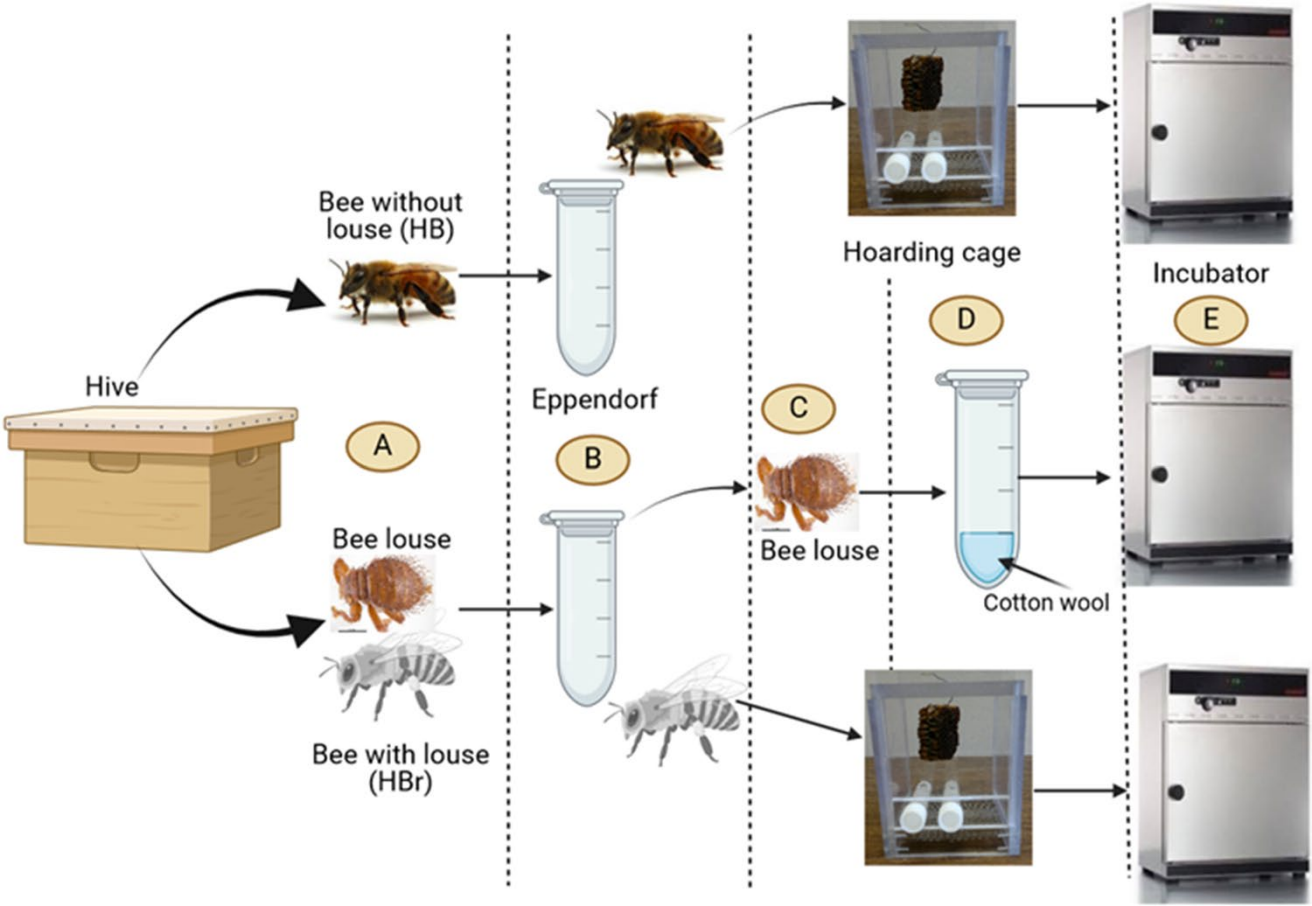
Steps involved in the sampling of honey bee workers carrying or not carrying the bee lice *Braula coeca* from honey bee hives and how they were kept in the laboratory prior to bioassays. Collection (**A**), separating bees carrying and those not carrying bee louse (**B**), removal of bee lice on bees (**C**) and placing into rearing Eppendorf containing moist cotton wool (**D**) and maintaining bees and bee lice in incubators (E) before bioassays. Broken vertical lines indicates the different sampling steps followed. Illustration created in https://biorender.com

Sampling and retrieval of honey bees with or without bee lice was conducted as shown on Fig. 1. Briefly, workers of the African Savannah honey bees *A. m. scutellata* carrying (HBr) and those not carrying (HB) the bee lice (*Braula coeca*) were sampled from four queenright colonies headed by naturally mated queens at the apiaries of the University of Pretoria South Africa (25°44′49"S, 28°15′40"E) that were maintained using standard apicultural practices (Williams et al. 2013; Yusuf et al. 2018). Individual worker bees (either HBr or HB) were quickly caught by their legs using entomological forceps and placed in a clean perforated Eppendorf tube (that allows for ventilation) (Fig. 1 step B) and transported to the laboratory.

In the laboratory, *B. coeca* were removed from the bees and placed in new clean Eppendorf tubes containing moist cotton wool (Fig. 1 steps C and D) for rearing. We found only one louse per bee. While the bees were placed in hoarding cages (one for bees carrying the bee louse and the other for those not carrying the bee louse) (Fig. 1 step D) and kept in incubators (Fig. 1 step E) set at 35 ˚C and 65% relative humidity until required for bioassays. Prior to bioassays, both honey bees and *B. coeca* were conditioned in the incubators for two hours in order to get them into the same physiological state.

### Choice of Worker Honey Bee Host by the Bee Lice (Braula Coeca) and the Sources of Olfactory Cues

**Fig. 2:**
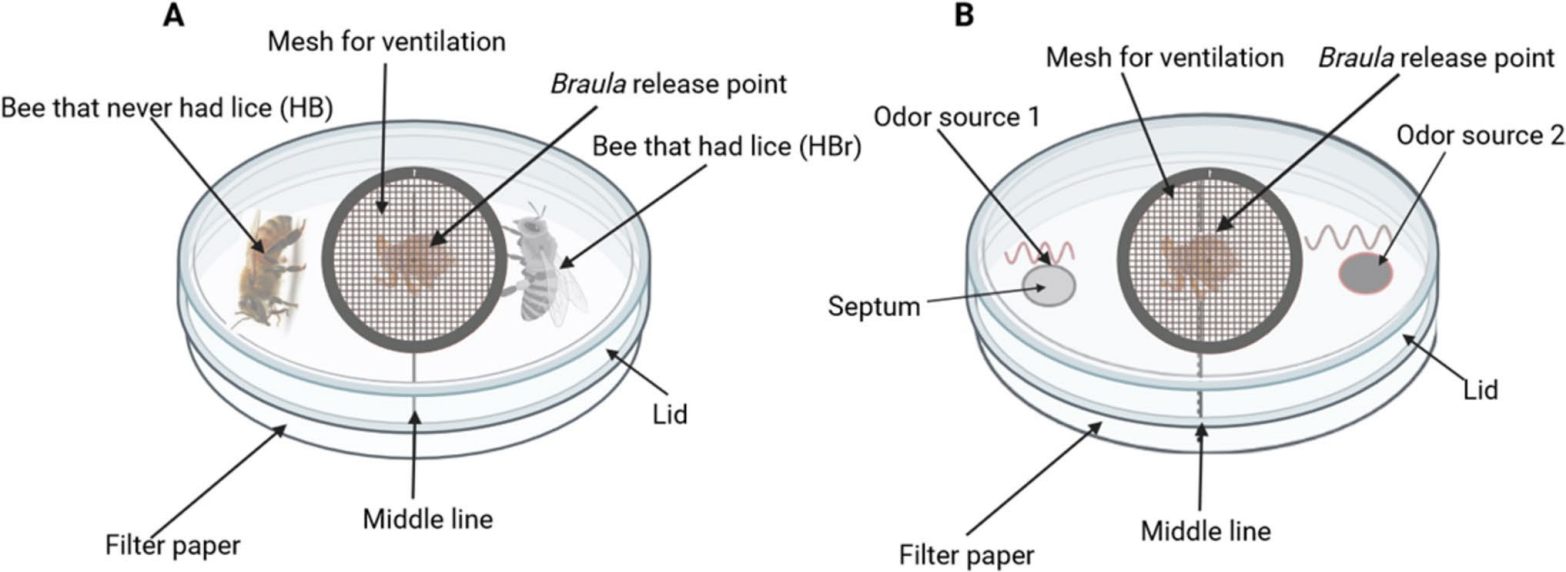
Petri dish bioassay arena set-up used to determine how *Braula coeca* choses its host between worker bees that carried the lice and those that did not carry it (A) when collected from the hive. A similar set-up (B) was used to test the different odor sources used by *B. coeca* for host selection. Middle line was a line drawn on the bottom of the Petri dish. Illustration made in https://biorender.com

In order to determine if the selection of a host by the bee louse (*B. coeca*) is by chance (random) or guided by visual and or olfactory cues, we conducted choice bioassays using modified 90 mm Petri dishes as arenas (Fig. 2). In Experiment I (Fig. 2A) to test if host choice is random, two honey bees (one previously carrying (HBr)) and the other not previously carrying *B. coeca* (HB) were uniquely marked on the thorax, wings or abdomen using water resistant non-toxic Schneider Maxx 270 Paint Marker (Schneider, Germany) and released in the arena, returned to the incubator and allowed five minutes to settle. After the settling time, *B. coeca* was introduced into the middle of the arena (along the middle line drawn on the bottom of the Petri-dish, Fig. 2A), returned back to the incubator and allowed 10 minutes to make a choice between the two worker bees. A choice was recorded as successful when the lice mount the bee. If no choice was made after 10 minutes, the experiment was terminated and recorded as unsuccessful (no mounting). A total of 80 workers (40 of which previously carried *B. coeca* and 40 that did not carry *B. coeca* previously) and 40 *B. coeca* were used for the bioassay. To test if the choice of host is based on olfactory cues, a similar set up like that in Experiment I was used (Fig. 2B). In order to eliminate visual cues from the worker bees, extracts from the mandibular glands (MDG) and cuticular hydrocarbons (CHC) from bees carrying (HBr) and not carrying (HB) the bee louse were made and used as olfactory cues in the bioassay.

Mandibular gland pheromones were extracted in 200 µL of dichloromethane (DCM) ChromSolv® grade for HPLC (Sigma Aldrich, USA) as described in Yusuf et al. (2015). While cuticular hydrocarbons were extracted by washing the cuticles of fours honey bees in one mL of *n*-hexane (Sigma Aldrich, Saint Louis, MO, USA) for two minutes. The choice of these sources of olfactory cues were informed by earlier studies (Kaschef 1959 and*,* Martin and Bayfield 2014 who reported responses of *Braula spp.* to bee pheromones and possession of similar CHC profiles to that of the bees) and results from Experiment I.

In Experiment II, 100 µL MDG or CHC extracts (odor sources) were loaded onto a rubber septum (type and manufacturer) that was previously sterilized by baking in an oven for three hours at 250 ˚C and the solvent allowed to evaporate prior to the assay. The test odours (MDG extracts against solvent (DCM), CHC extracts against solvent (*n*-hexane) and MDG extracts against CHC extracts) were provided in the bioassay arena (Fig. 2B) and *B. coeca* introduced to the arena, allowed five minutes to settle and the observed for ten minutes. A choice was recorded when *B. coeca* climbed and stays on the septa. Fifty (50) *B. coeca* were used for each odor combination test.

### Chemical Analysis of Mandibular Gland (MDG) Extracts and Cuticular Hydrocarbons (CHC) from Honey Bees and Braula Coeca

To determine the chemical composition of MDG and CHC extracts used in Experiment II, and those of MDGs from worker bees not carrying *B. coeca* (HB)*,* and CHC profiles of *B. coeca,* the extracts were analyzed using a Gas Chromatograph Flame Ionization Detector (GC-FID) and GC coupled Mass Spectrometer (GC–MS).

*Mandibular gland extracts*: Heads (MDG) extracts were analyzed following the method described in Yusuf et al. (2015). Briefly, one (1) μL of the derivatized extract was injected in split less mode into an Agilent 6890 series GC-FID fitted with a 25 m × 0.32 mm × 0.22 μm HP1-MS column (Agilent J&W, Santa. Clara, USA). The carrier gas was helium at a flow rate of 1 mL/min; and oven temperature programmed as follows: 60 °C for 1 min, then heated at 50 °C/min to 110 °C, then 3 °C/min to 220 °C, and then held at 220 °C for 10 min. Five of the major components from mandibular glands of honeybees, that had been shown to elicit both behavioral and physiological responses namely; 9-oxo-2(*E*)-decenoic acid (9-ODA), 9-hydroxy-2(*E*)-decenoic acid (9-HDA), methyl *p*-hydroxybenzoate (HOB), 10-hydroxy decanoic acid (10-HDAA) and 10-hydroxy-2(*E*)-decenoic (10-HDA), were identified based on comparison with retention times of synthetic standards. Quantification was achieved, by comparing the relative mass ratios (RMR) of each compound in a standard solution mixture containing 1 mg of each in 4 mL DCM relative to the RMR of 1 mg *n-*tetradecane.

#### Chemical analysis of Cuticular hydrocarbons

In order to determine the CHC profiles from *B. coeca,* ten (10) individuals were extracted in 250 μL of *n*-hexane for two (2) minutes. This and the CHC extracts from worker bees carrying *B. coeca* (HBr) and those not carrying (HB) were analyzed on a Shimadzu QP-2010 SE GC–MS (Shimadzu Corporation, Japan) equipped with a Rtx-5MS 30 m, 0.25 mm ID, 0.25 μm column (Restek Corporation, Bellefonte, PA, USA) as follows. One (1) μL of each extract was injected in the split less mode at 250 ˚C with Helium as a career gas at a flow rate of one (1) mL/min. The oven was programmed at 120 ˚C, ramped at 15 ˚C/min to 310 ˚C, and held for ten (10) minutes, while the Ion source, and interface temperatures were set at 200 and 300 ˚C respectively. The MS was operated in the electron ionization mode at 70 eV, scan speed of 2500 per 0.30 s between 50 to 700 m/z. Compounds were identified by comparing their mass spectra with those from commercial libraries NIST 09 and Wiley 09, and using a mixture of C13 – C40 straight chained alkanes. Quantification was achieved using an internal standard made up of 0.066 μg per μL C21 (*n*-heneicosane) that was added to the sample prior to analysis.

### Bioassay with Synthetic Mandibular Gland Pheromones

To identify the specific compounds used by *B. coeca* to choose its host, a dose response bioassay (using a similar set-up in Fig. 2B) was conducted with the individual mandibular gland pheromone components (9-ODA and 10-HDAA) that were different among honey bee workers carrying (HBr) and those not carrying *B. coeca* (HB)*.* The doses used were 0.50, 1.0, 2.5 and 5.0 μg for 9-ODA, and 0.9, 2.0, 4.0 and 8.0 μg for10-HDAA.

#### Chemicals

All reagents and chemical standards used were of analytical grade at a purity of > 98% obtained from Sigma-Aldrich, while 9-ODA was synthesized by Glaxo Chemicals (UK).

### Statistical Analyses

Data for the choice bioassays were analyzed using a one-sample Chi-Square (χ ^2^) tests where the number of *B. coeca* responding to or not responding to the test odors source were compared. Non-responders were excluded from the statistical analyses to prevent bias as they do not contribute to the test. Because the data from MDG pheromones were not normally distributed, Mann–Whitney U-test was used to test for differences in the amounts of the five mandibular gland components. Furthermore, a Kruskal–Wallis ANOVA test for multiple comparisons was used to compare variability among the mandibular gland components between bees carrying *Braula* and those not carrying *Braula*. All statistical analyses were performed using the software STATISTICA 11 (Statsoft, USA).

## Results

### Choice of Honey Bee Host by Braula coeca

**Fig. 3:**
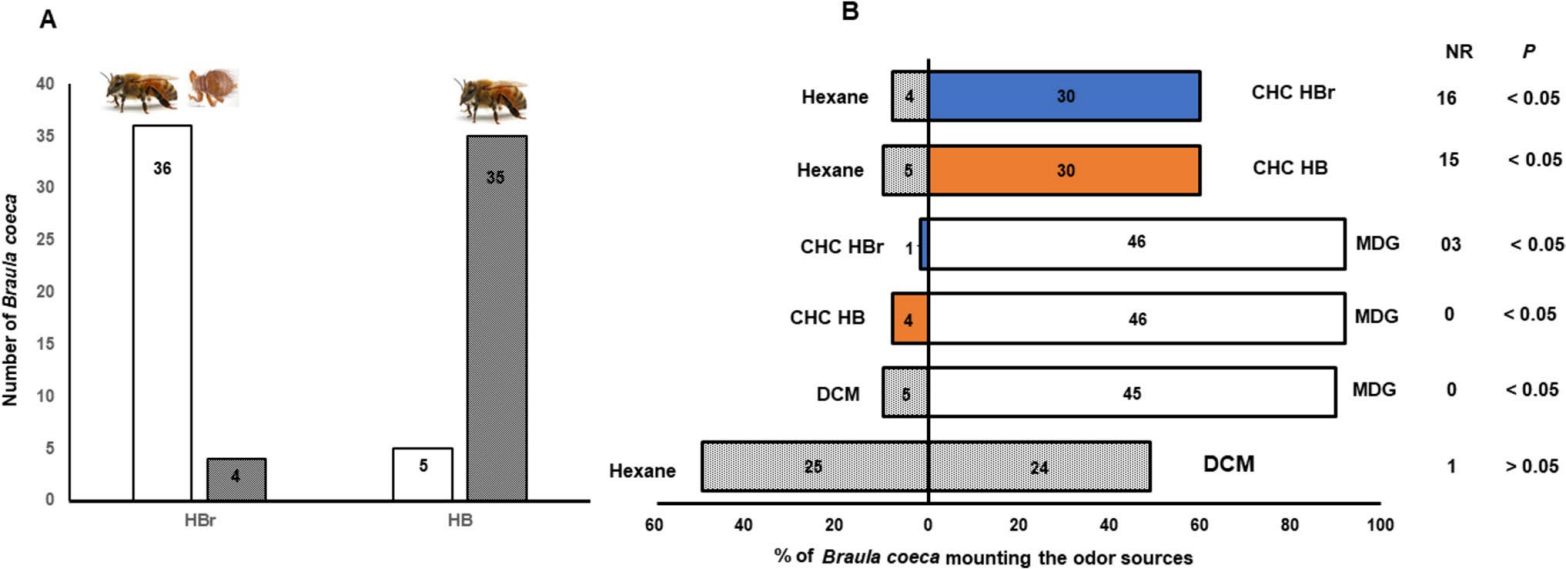
Remounting of honey bee workers previously carrying (HBr) and those that did not carry (HB) the bee lice *Braula coeca* (**A**). Open bars represent successful remounting while checked bars represent unsuccessful mounting. Preferences (**B**) of *B. coeca* to extracts (odors) of Mandibular glands (MDG) open bars, cuticular hydrocarbons (CHCs) from HBr blue, CHCs from HB (orange), and solvent controls (checked bars). DCM = dichloromethane, NR = non-responding *B. coeca* and P = p values. Numbers inside the bars represent the *B. coeca* that responded to the treatment

*Braula coeca* successfully re-mounted honey bee workers that previously carried them (HBr) but not worker bees that had not previously carrying them (HB) (Fig. 3A, χ^2^ = 281.7, P < *0.05*). When choices of extracts were provided, *B. coeca* showed preferences for mandibular glands over the solvent control (χ^2^ = 22.9, P < *0.05*) and cuticular hydrocarbons from HBr (χ^2^ = 32.3, P < *0.05*) and HB (χ^2^ = 25.5, P < *0.05*) (Fig. 3B).

### Mandibular Gland (MDG) and Cuticular Hydrocarbon (CHC) Profiles from Honey Bees and Braula Coeca

#### Mandibular Gland Compounds

**Fig. 4:**
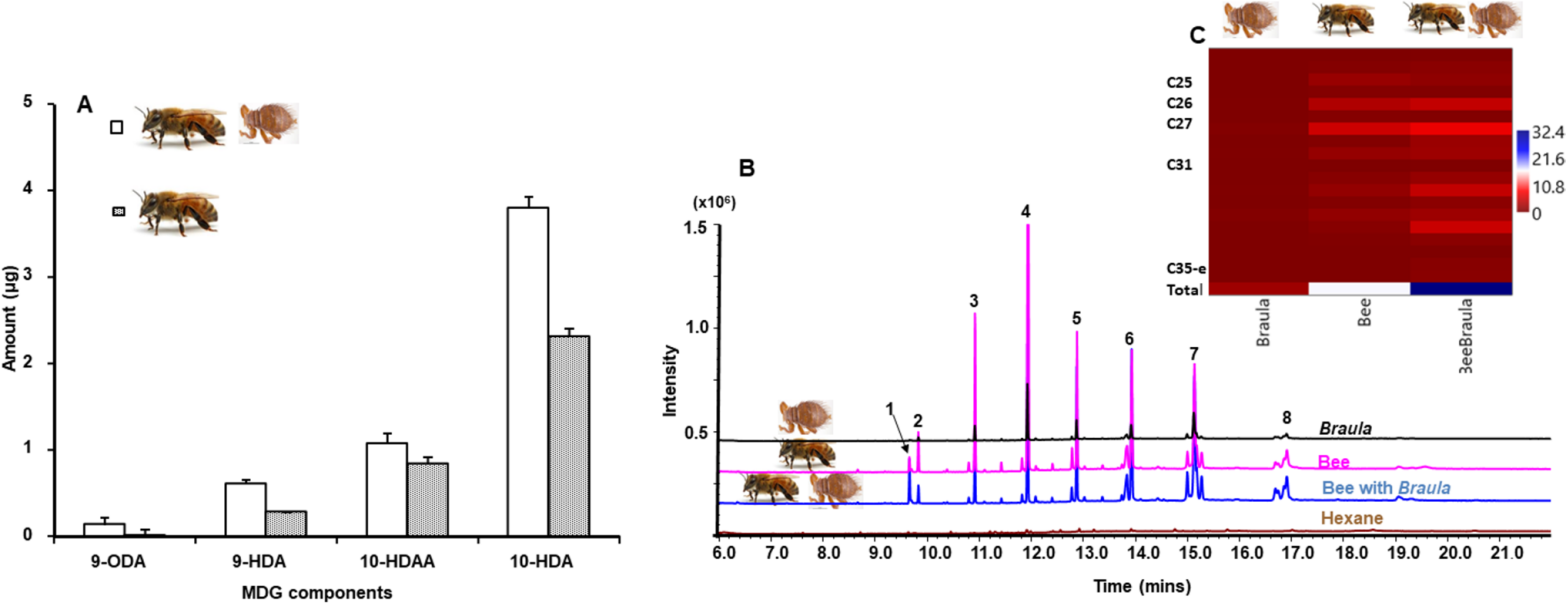
**A** Amount of mandibular gland pheromones 9-oxo-2(*E*)-decenoic acid (9-ODA), 9-hydroxy-2(*E*)-decenoic acid (9HDA), 10-hydroxy decanoic acid (10-HDAA) and 10-hydroxy-2(*E*)-decenoic(10-HDA) in µg from head extracts of honey bee workers carrying *Braula* (open bars) and those not carrying (checked bars). **B** Total Ion Chromatogram (TIC) of Cuticular Hydrocarbons (CHC, 1 = Eicosanol, 2 = *n-*Tricosene, 3 = *n-*Pentacosane, 4 = *n-*Hexacosane, 5 = *n-*Heptacosane, 6 = Hentriacontane, 7 = *n-*Dotriacontane, 8 = Pentatriacontene) from *Braula coeca* (black), honey bee not carrying *B. coeca* (pink), honey bee carrying *B. coeca* (blue) and hexane blank (brown), Fig. 4C The amount of CHCs from *Braula,* honey bee and honey bee carrying *B. coeca*

Quantitatively, bees carrying (HBr) *B. coeca* had higher total amounts of pheromones with a mean of 6.02 ± 0.59 µg per bee, compared to 3.62 ± 0.48 µg for bees not carrying (HB) *B. coeca* (Fig. 4A, Table S1). The relative amounts of the individual components 9-ODA, 9-HDA and 10-HDAA were significantly higher in bees carrying *B. coeca* than in those not carrying (P < 0.05, Mann–Whitney U- Test, Fig. 4A). Mandibular gland pheromones between HBr and HB sampled from the four experimental colonies except 10-HDAA were significantly different (KWA: H = 5, N = 106, P < *0.05*).

#### Cuticular Hydrocarbon Profiles

The cuticular hydrocarbon profiles of *B. coeca* and worker bees carrying and those not carrying *B. coeca* are qualitatively identical (Fig. 4B) and is made up of hydrocarbons (CHC) with chain lengths between C19 – C35. The main components in the profiles were *n*-pentacosane, *n*-hexacosane, *n*-heptacosane, *n*-hentriacontane and *n*-dotriacontane (Fig. 4C).

### Bioassay with Synthetic Mandibular Gland Pheromones

**Fig. 5:**
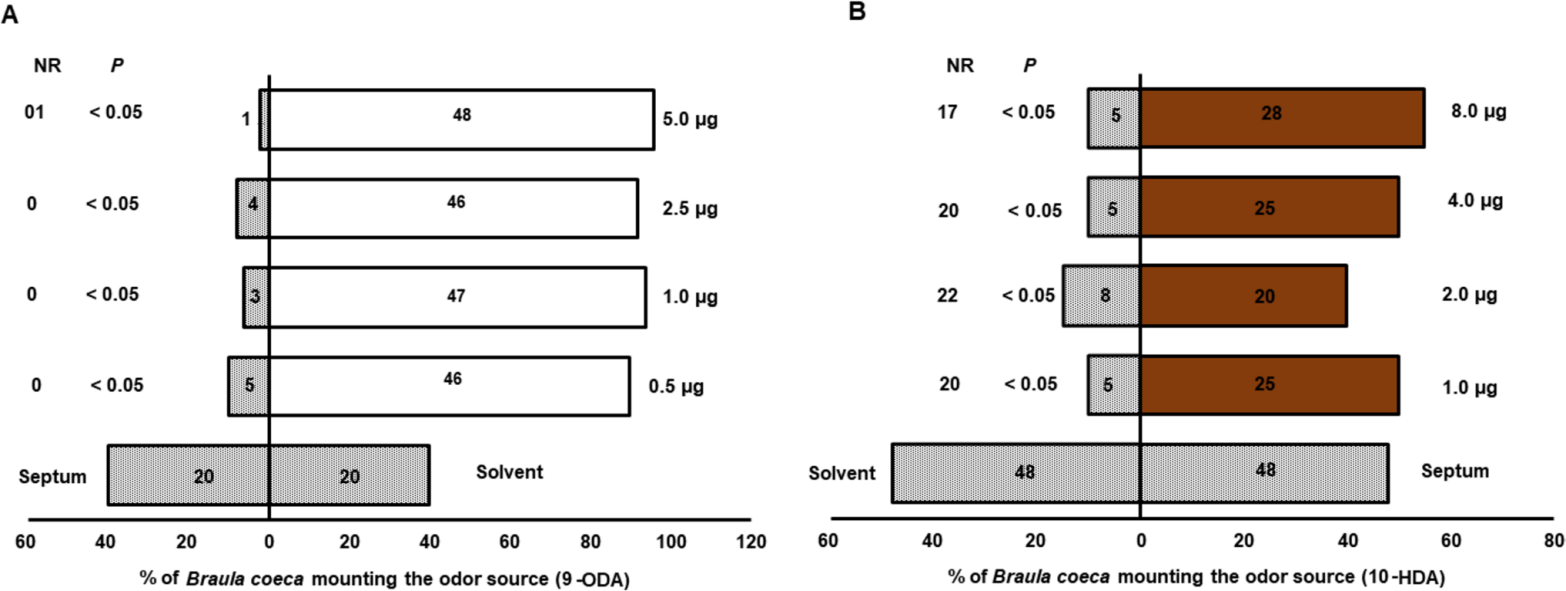
Responses of *Braula coeca* to different doses of the queen substance 9-ODA (**A**) and worker substance 10-HDA (**B**). Checked bars represent solvent control, open bars represent 9-ODA and brown bars, 10-HDA. Numbers in the bars represent individual *B. coeca* responding to the test odors

*Braula coeca* responded more to different doses of the queen bee substance (9-ODA) than to the solvent control (Kolmogorov-Smirov test, P < 0.05, Fig. 5A). The responses were between 90 and 96% at the highest dose of 5.0 µg per µL. While the response of *B. coeca* to the worker substance 10-HDA were different (Kolmogorov-Smirov test, P < 0.05) against the control and varied between doses and were only between 40 and 55% at the highest dose of 8.0 µg (Fig. 5B).

## Discussion

Using re-mounting bioassays, we have shown that the bee louse *B. coeca* preferred worker honey bees that initially carried it in the hive and used olfactory cues to select its host in order to enhance the chances of getting food. Further, we have shown that visual cues are not essential in this interaction between *B. coeca* and its host the honey bees due to the uniqueness of the hive environment in which olfaction takes precedence over other sensory cues.

The cues used by the bee lice are from the mandibular glands specifically the queen substance 9-ODA and worker substance 10-HDA not those from the cuticle. Workers that carried *B. coeca* on their thoraces have significantly higher concentrations of mandibular gland pheromones (about two-fold) more in comparison to those not carrying *B. coeca* in the hive. This indicates that the fly is able to detect host workers through their pheromones especially those that produce more pheromones than their co-workers. Thus, confirming findings based on the sensory physiology of *B. coeca* by Kaschef (1959) suggesting that, the bee louse have receptors that are tuned to detect host specific pheromones in the hive where olfactory cues play an important role in communication.

The use of olfactory cues by arthropod pest living in the hive has been demonstrated and or suggested for the parasitic mite *Varroa destructor* which have the ability to detect relative concentrations of geraniol and nerolic acid from the Nasonov glands of older workers, and use them as cues to avoid older bees in preference to nurse bees (Pernal et al 2005). Pernal et al. (2005) also mentioned that cues of a volatile nature from newly emerged bees serve as the initial stimulus, (long range cues) to disperse the mites in search of a host before they are guided by allomonal cues (short range cues) from older workers to locate nurse bees. It was also found that methyl esters, ethyl palmitates and methyl linolenate extracted from cuticles of drone (Le Conte et al. 1989) and worker larva of *A. m. linguistica* (Trouiller et al. 1991) serve as pheromones and kairomones responsible for attracting the parasitic *Varroa* mite to the brood. The small hive beetle *Aethina tumida* also have the ability to detect pheromone odors from honey bees (Torto et al. 2005) and uses chemical mimicry through cuticular hydrocarbons to masks itself in the hive (Amos et al. 2022) and receive protein rich food from workers (Langlands et al 2021). In contrast, *B. coeca* did not show preferences for cuticular hydrocarbons but possessed the same CHC profiles, indicating that it does not use these cues for host detection, rather for camouflage within the hive (Martin and Bayfield 2014).

Our bioassays and pheromonal data provide an explanation and support for the observations made by Smith and Caron (1984) in field and nuclear colony experiments where they showed that *B. coeca* prefers queens over drones. We found differences in the composition of pheromones between workers carrying and those not carrying *B*. *coeca*, with those carrying possessing more of the queen (9-ODA) and worker substances 10-HDA, making them smell or have profiles similar and closer to those of the queen (Crewe and Velthuis 1980; Zheng et al. 2010). *Braula coeca* also responded to 9-ODA and 10-HDA in bioassays, although their preference for the queen substance was more than that for the worker substance, which could explain why the bee lice are attracted more to queens, followed by workers (Smith and Caron 1984). Indeed, there exist qualitative differences in MDG secretions of workers that are dominated by two fatty acids 10-HDAA and 10-HDA (Plettner et al. 1993) and those of the queen dominated by 9-ODA (queen substance) (Slessor et al. 1988) which result in differential treatment by other workers and represent a different status within the colony (Schäfer et al. 2006; Zheng et al. 2010). The possession of queenlike pheromone components makes workers dominant (Crewe and Velthuis 1980; Zheng et al. 2010), and these workers are more likely to be fed by other bees (Crailsheim 1991; Schäfer et al. 2006). Hence, attaching to the thorax of a bee that smells more would increase the chances of obtaining food for the bee lice against attaching itself to a worker that does not possess this signal and is less likely not to receive food or attention from other workers during trophallactic exchange. Attraction of *B. coeca* to workers with different quantities (amount) of mandibular gland pheromones suggests a method of detecting and responding to host kairomones by an arthropod pest associated with the bee hive similar to behavior exhibited by *V. destructor* (Pernal et al 2005) and *A. tumida* (Torto et al. 2005). Given the differences in the pheromonal cues from worker honey bees we conclude that, as a result of co-evolution and adaptation, *B. coeca* has developed mechanisms that enable it to discriminate between potential and ideal host workers via specific olfactory cues as honey bees are the only known host of *B. coeca.*

In summary, we provide evidence that *B. coeca* eavesdrops on their host’s pheromones to make choices between individual workers. There is a likelihood of the bee louse exploiting a similar mechanism in other honey bee subspecies, showing similar pheromonal patterns among workers and queens. The observed host preferences are likely to affect the louse's survival and abundance. For instance, in sub-species where only the queens produce queen-like pheromones, *Braula spp*. would have fewer suitable hosts and consequently lower prevalence, whereas in African sub-species like *A. m. capensis* with worker individuals that mimic the queen, one would expect *Braula spp.* to have higher prevalence and chances of survival.

### Supplementary Information

Below is the link to the electronic supplementary material.

Supplementary file1 (DOCX 15 KB)
