# Supplementary material for: A Hitchhiker’s Ride: The Honey Bee Louse Braula Coeca (Diptera: Braulidae) Selects its Host by Eavesdropping
Source: J Chem Ecol. 2024 Feb 23;50(5-6):214–21. doi: 10.1007/s10886-024-01481-2 (PMC11233430; doi:10.1007/s10886-024-01481-2)
Supplement: Supplementary file 1 — Supplementary file1 (DOCX 15 KB) [file 10886_2024_1481_MOESM1_ESM.docx]

**Supplementary material**

Table S1-Mean amounts of mandibular gland pheromone components from bees carrying (HBr) and those not carrying (HB) the bee lice *Braula coeca*

|  | **Amount (ug) ± SE** | | | | |
| --- | --- | --- | --- | --- | --- |
| **Bee** | **HOB** | **9-ODA** | **9-HDA** | **10-HDAA** | **10-HDA** |
| HBr (N = 64) | 0.4 ± 0.07 | 0.14 ± 0.04 | 0.62 ± 0.11 | 1.07 ± 0.13 | 3.80 ± 0.41 |
| HB (N = 42) | 0.18 ± 0.07 | 0.01 ± 0.01 | 0.28 ± 0.07 | 0.85 ± 0.10 | 2.31 ± 0.40 |

HOB is methyl p-hydroxybenzoate, 9-ODA is 9-oxo-2 (E)-decenoic acid, 9-HDA is 9-hydroxy-2-decenoic acid, while 10-HDAA and 10-HDA are the honey bee worker specific mandibular gland pheromones, 10-hydroxy-2 (E)-decenoic acid and 10-hydroxydecanoic acid respectively.
